# The Effect of Polyphenols on Working and Episodic Memory in Non-pathological and Pathological Aging: A Systematic Review and Meta-Analysis

**DOI:** 10.3389/fnut.2021.720756

**Published:** 2022-01-26

**Authors:** Karin de Vries, Evelyn Medawar, Aniko Korosi, A. Veronica Witte

**Affiliations:** ^1^Swammerdam Institute for Life Sciences, University of Amsterdam, Amsterdam, Netherlands; ^2^Department of Neurology, Max Planck Institute for Human Cognitive and Brain Sciences, Leipzig, Germany; ^3^Faculty of Philosophy, Berlin School of Mind and Brain, Humboldt-Universität zu Berlin, Berlin, Germany; ^4^Clinic for Cognitive Neurology, University Medical Center Leipzig, Leipzig, Germany

**Keywords:** polyphenol, RCT—randomized controlled trial, aging, episodic memory, working memory

## Abstract

Life expectancy steadily increases, and so do age-associated diseases, leading to a growing population suffering from cognitive decline and dementia. Impairments in working memory (WM) and episodic memory (EM) are associated with an increased risk of developing dementia. While there are no effective pharmacological therapies to preserve or enhance cognition and to slow down the progression from mild memory complaints to dementia so far, plant-based nutrients including polyphenols have been suggested to exert beneficial effects on brain aging. This review studies whether supplementary polyphenols are effective in preserving or enhancing memory in both non-pathological and pathological aging, and whether there are polyphenol efficiency differences between WM and EM. A systematic literature search was conducted and 66 out of 294 randomized clinical trials with 20 participants or more per group, aged 40 years or older were included. These covered a daily intake of 35–1,600 mg polyphenols, e.g., flavonols, flavonoids, isoflovones, anthocyanins, and/or stilbenes, over the course of 2 weeks to 6.5 years duration. In total, around half of the studies reported a significantly improved performance after polyphenol administration compared to control, while three studies reported a worsening of performance, and the remainder did not observe any effects. According to pooled WM and EM meta-analysis of all memory outcomes reported in 49 studies, overall effect size for WM and EM indicated a significant small positive effect on EM and WM with similar estimates (*b* ~ 0.24, *p* < 0.001), with large study heterogeneity and significant Funnel asymmetry tests suggesting a positivity bias. These results remained similar when excluding studies reporting extremely large positive effect sizes from the meta-analyses. While *Ginkgo biloba* and isoflavones did not show benefits in subgroup meta-analyses, those suggested some effects in extracts containing anthocyanins, other flavonoids and resveratrol, again potentially resulting from publication bias. To conclude, a systematic review and meta-analysis indicate that short- to moderate-term polyphenol interventions might improve WM and EM in middle-to older aged adults, however, publication bias in favor of positive results seems likely, rendering definite conclusions difficult. Future studies with larger, more diverse samples and sensitive monitoring of cardiovascular, metabolic and beginning brain pathologies as well as longer follow-up are needed to better understand the impact of age, (beginning) pathologies, gender, and long-term use on polyphenol action.

## Introduction

Due to economic, social, and health care developments, the life expectancy of people in all regions of the world is increasing. As a consequence, the proportion of people aged 65 or older is expected to rise from 1 in 11 people in 2019 to 1 in 6 people in 2050 (1). Aging is associated with deteriorating health, including brain health. With aging, for example, pro-inflammatory activity and less efficient anti-oxidative mechanisms lead to higher burden of neuroinflammation and oxidative damage in the brain (2). Moreover, an increase in neurodegeneration (i.e., loss of neurons) and a reduction in neurogenesis (i.e., formation of new neurons) occur with aging and negatively affect the neuronal plasticity of the brain (3, 4). These brain alterations are thought to underly cognitive decline and memory impairment, a key symptom of dementia such as in Alzheimer's disease (4). As the number of people with dementia could likely expand to 132 million people by 2050, causing extreme social and individual costs (5–7), healthy brain aging constitutes a global challenge. However, currently, there are no effective pharmacological therapies to preserve or enhance cognition in older age (8, 9).

While unhealthy lifestyle can accelerate the process of cognitive decline during aging, health-promoting lifestyle factors such as physical activity and nutrition might slow down the trajectory of cognitive decline (10). Therefore, the interest in studying the influence of polyphenols on cognitive functioning is rising. Polyphenols are micronutrients that are found in plant-based foods (4). There are several subclasses of polyphenols, of which flavonoids, stilbenes, and phenolic acids are examples (11). Multiple phenol groups per molecule characterize polyphenols, but the chemical properties of different polyphenols are heterogeneous (12). Studies suggest that polyphenols can cross the blood-brain barrier and affect aging processes due to their anti-inflammatory, antioxidant, and neuroprotective properties (2, 13). In the past ~15 years, numerous animal experiments and human studies have investigated whether polyphenols enhance cognitive performance or prevent age-related brain pathologies, yet the level of scientific evidence and clinical efficiency in humans still remain unclear [reviewed e.g., in (14–16)].

Therefore, we aimed to perform a systematic review whether polyphenols are effective in preserving or enhancing memory in (non-)pathological human aging. We decided to consider results from randomized-controlled trials (RCT) only, as this study design is the most ideal to demonstrate a causal relationship between an intervention and an effect and is an important step toward evidence-based therapies (17). We focused on the memory system because (1) memory decline may underlie changes in other cognitive functions and (2) memory complaints are reported first in the preclinical trajectory of cognitive decline, sometimes already 16 years before diagnosis (18, 19). Two common types of dementia, namely frontotemporal dementia and Alzheimer's disease, are characterized by early-stage deficits in working memory (WM) and episodic memory (EM) respectively, underlining (partly) independent neuronal underpinnings of the two memory processes (20). WM is required to direct attention and manipulate information that is stored in short-term memory (21, 22) and EM allows to learn, store, and retrieve information about personal experiences (22, 23). In addition, impairments in either WM and EM are associated with an increased risk of developing dementia (24, 25). We therefore studied both WM and EM and asked whether polyphenols are effective in preserving or enhancing memory in (non-)pathological human aging.

## Methods

### Literature Search

**Figure 1:**
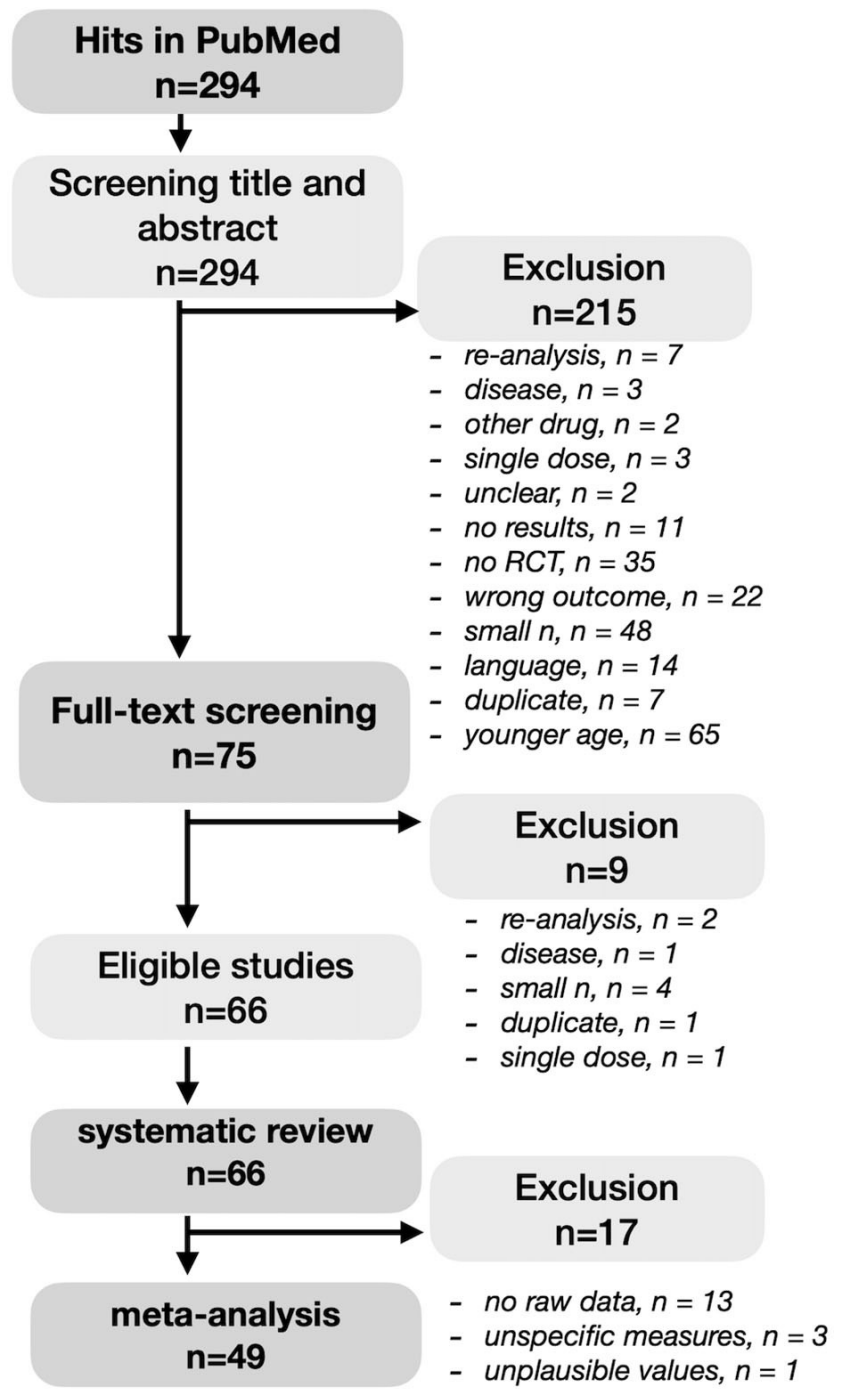
PRISMA diagram of the systematic literature search.

A literature search was conducted in PubMed in August 2021 (Figure 1). We decided to The search term ((((cognitive AND blueberry AND (humans[Filter])) OR (cognitive AND gingko AND (humans[Filter]))) OR (cognitive AND curcumin AND (humans[Filter]))) OR (cognitive AND polyphenol AND (humans[Filter]))) OR (cognitive AND flavonoids AND (humans[Filter])) together with the filter “clinical trial” or “clinical study” or “RCT” resulted in 294 hits. All articles were screened on the following inclusion criteria: (1) RCT study design, (2) administration of polyphenols or polyphenol-rich extracts or supplements, (3) an included measure of working or episodic memory, (4) a sample size of at least 20 participants per group with available follow-up data (i.e., completers), and (5) the participants included in the sample had to be at least 40 years old. Exclusion criteria were non-English articles, single dose trials, severe disease of participants (such as depression, multiple sclerosis, cancer) as well as duplicates or re-analyses of previously published trials. A study outcome measures was identified as memory performance-related and grouped into EM and WM, respectively, based on the author's descriptions and/or by inspecting the description of the used tests in the literature. No self-reported memory measures were evaluated. Note that several neuropsychological tests used in dementia patients, such as the ADAS-cog, usually do not provide raw memory subscale values. These studies were included in the systematic review but excluded from meta-analyses due to the lack of specificity regarding memory functions. A cutoff number of minimally 20 participants per group was used to decrease the likelihood of a type II error, which can skew the results toward not finding a (small) effect that is truly there. Considering the age range definition, cognitive decline already starts from young adulthood, but by middle age, from around 40, neuronal volume shrinkage in both white and gray matter becomes more apparent (26, 27). According title and abstract screening, as well as occasionally briefly consulting full-texts, resulted in 75 RCTs with the majority of studies excluded due to wrong population, wrong micronutrient, no memory measure, no RCT, younger age, small sample. During the full-text screening, another nine articles were excluded based on re-analysis (*n* = 2), non-dementia disease (*n* = 1), small sample size (*n* = 4), duplicate (*n* = 1), and single dose study (*n* = 1). Full-text screening eventually resulted in 66 articles that were included in the systematic review. Effect sizes could be derived or calculated from 49 articles and included in a meta-analysis. Due to unavailable raw value information, effect sizes could not be retrieved from 17 studies.

As all included articles provided relatively large sample sizes per group (>*n* = 20) and all were carefully checked for following a randomized trial design being regarded as the gold standard, we refrained from applying additional quality evaluation tools.

### Included Polyphenols and Their Effects on Memory

The identified studies investigated either polyphenol-rich plant extracts such as berries, cherries, grapes, pomegranate, green tea, and *Ginkgo biloba*, cocoa flavanols, curcumin, *Pinus radiata* bark, spearmint extract, soy isoflavonol, or rather purified polyphenols such as resveratrol (see Table 1 for a more detailed overview). In addition, studies included different control conditions, either placebo or no treatment, or a lower dosage of the polyphenol treatment, or an alternative drug, such as rivastigmin or donezepil for treatment of Alzheimer's disease symptoms or alternative substances not containing polyphenols. Results were further reviewed according to these categories and reported WM and EM outcomes, respectively.

**Table 1:** Details of the 66 included studies in the review.

**Study no.#**	**First author**	**Publication year**	**Journal**	**Population**	**Age range (years)**	**Name of the examined extract, polyphenol or sublass (referred to as main or effective ingredient by the authors)**	**Intervention, amount of polyphenols**	**Control**	**Duration of intervention/ control period**	**n**	**Cognitive tests related to working or episodic memory performance**	**Any significant outcome (**°**none, + yes positive; - yes negative)**	**Significant outcomes in working (WM) or episodic memory (EM), or both**	**included in metaanalysis**	**Reference**
**1**	Ahles S	2020	Nutrients	healthy adults (overweight-obese, BMI25-35)	40-60	*anthocyanins*	90 mg Aronia melanocarpa, 150 mg Aronia melanocarpa (18%anthocyanins)/day	maltodextrin	24weeks	102 (97 completed)	Stroop	°		yes	(28)
**2**	Basaria S	2009	J Endocrinol Invest	perimenopausal women	46-76	*isoflavones*	160 mg of total isoflavones (96 mg aglycones)/day	casein	12 weeks	93 (84 completed, 46+38)	TMTB	°		no (unplausible values)	(29)
**3**	Beck SM	2016	Hum Psychopharmacol	healthy adults, SMI	50-65	*ginkgo biloba*	240 mg, 22-27% ginkgo flavones/day	placebo (unclear)	8 weeks	75 (61−30,31 completed)	task-set switching, delayed response task, prospective memory	+	WM	yes	(30)
**4**	Bensalem J	2019	J Gerontol A Biol Sci Med Sci	healthy adults	60-70	*anthocyanins*	600 mg/day	placebo (unclear)	24 weeks	215 (190 completed)	Cambridge Neuropsychological Test Automated Battery (CANTAB): PALTEA, VRMFR, VRMR, SSP, reverse SSP	+	EM	yes	(31)
**5**	Burns NR	2006	Hum Psychopharmacol	healthy adults	55-79	*ginkgo biloba*	120mg/day	placebo (unclear)	12 weeks	93	test battery including wordlist, Stroop, digit span	+	EM	yes	(32)
**6**	Carlson JJ	2007	J Am Diet Assoc	healthy adults	65-84	*ginkgo biloba*	3xcapsules, =160 mg ginkgo biloba, 68 mg gotu kola, and 180 mg decosahexaenoic acid, bioflavonoid concentrate (100 mg) and vitamin A (300 IU)/day	identical capsules (gelatin, glycerin, soybean oil, yellow beeswax, lecithin, corn oil, natural caramel color, and maltodextrin)	4 months	90 (78 completed)	benton visual retention, word list learning	-	EM	yes	(33)
**7**	Casini ML	2006	Fertil Steril	postmenopausal women	>12 months after menses absence	*isoflavones*	600mg/day (60mg/day isoflavones)	identical placebo (unclear)	6 months	78 (76 completed, cross-over)	digit span	+	WM	yes	(34)
**8**	Cieza A	2003	Fortschr Med Orig	healthy adults	50-65	*ginkgo biloba*	240mg/day	placebo (unclear)	4 weeks	66 (66 completed)	different psychological tests including Stroop, digit span	°		yes	(35)
**9**	Cox KH	2015	J Psychopharmacol	healthy adults	60–85	*curcuminoids*	400 mg/day Longvida® Optimized Curcumin (containing approximately 80 mg curcumin)	dextrin + yellow food powder	4 weeks	61 (60 completed)	parallel versions of tasks from the Computerised Mental Performance Assessment System	+	WM	yes	(18)
**10**	Cox KHM	2020	Nutrients	healthy adults	50-80	*curcuminoids*	400mg/day Longvida (80mg/day curcumin)	dextrin + yellow food powder	12 weeks	89 (79 completed)	Serial substractions, Virtual Morris Water Maze (vMWM)	+	WM	yes	(36)
**11**	Desideri G	2012	Hypertension	MCI	64-82	*cocoa flavanols*	high (HF: ≈990 mg flavanols/day) or intermediate (IF: ≈520 mg flavanols/day	low level (LF: ≈48 mg flavanols/day)	8 weeks	90	Verbal fluency, TMTA, TMTB	+	WM	yes	(37)
**12**	Dodge HH	2008	Neurology	healthy adults	>85	*ginkgo biloba*	3x80mg, =240mg/day (24% flavone glycosides)	unclear	42 months	134 (118 completed)	Cerad verbal learning	°		no (no raw measures available)	(38)
**13**	Evans HM	2017	Nutrients	cognitively intact post-menopausal women	45–85	*resveratrol*	150mg/day	placebo (unclear)	14 weeks	80 (72 completed)	Cambridge Semantic Memory Battery; Rey Auditory Verbal Learning Test (RAVLT); Double Span Task	+	EM	yes	(39)
**14**	Fournier LR	2007	J Nutr Health Aging	postmenopausal women	48-65	*isoflavones*	soy milk or soy supplement (70/72mg isoflavones)	cow milk, placebo supp.	16 weeks	79	Stroop, pattern recognition, benton, etc.	-	WM	no (no raw measures available)	(40)
**15**	Furlong ON	2020	Eur J Nutr	postmenopausal women	44-63	*isoflavones*	60mg/day or 35mg/day (in 350ml soy drink)	10mg/day	12 weeks	115 (101 analysed)	spatial working memory, spatial span, pattern recognition memory, 5-choice reaction time, and match to sample visual search	°		yes	(41)
**16**	Gavrilova SI	2014	Int J Geriatr Psychiatry	MCI	>55	*ginkgo biloba*	240mg per day	placebo (unclear)	24 weeks	160(145 analysed)	TMT-B	+	WM	yes	(42)
**17**	Gleason CE	2015	J Alzheimers Dis	AD	>60	isoflavones	100mg/day	maltodextrin	6 months	65 (59 completed)	verbal and visuospatial memory, Benton retention task, figure recall, TMT, etc)	°		yes	(43)
**18**	Hartley DE	2004	Nutr Neurosci	postmenopausal women	51-66	*ginkgo biloba*	320mg Gincosan/day (120mg ginkgo + 200mg ginseng)	unclear	12 weeks	70 (57 completed)	verbal and visual memory, CANTAB, delayed matching to sample test	°		yes	(44)
**19**	Henderson VW	2012	Neurology	perimenopausal women	45–92 years	isoflavones	25 g/day of isoflavone rich soy protein(contained 91 mg aglycone weight isoflavones (154 mg total isoflavone equivalents) composed of genistein (52 mg aglycone equivalents), daidzein (36 mg aglycone equivalents), and gly- citein (3 mg aglycone equivalents))	milk protein placebo	2.5 years	350 (313 completed)	Immediate recall, faces 2 delayed recall	+	EM	yes	(24)
**20**	Herrlinger KA	2018	J Altern Complement Med	healthy adults	50-70	chlorogenic acid	900mg or 600mg spearmint extract/day	powder with 0 mg/ day spearmint extract	90 days	90 (87 completed)	CDR battery	+	WM	yes	(45)
**21**	Herrschaft H	2012	J Psychiatr Res	mild-moderate AD	>50	*ginkgo biloba*	240mg/day	unclear	24 weeks	410 (404 analysed)	SKT, CGIC, verbal fluency	+	WM	no (no raw measures available)	(46)
**22**	Ho SC	2007	Menopause	postmenopausal women	55-76	isoflavones	80 mg/day soy-derived isoflavones	identical appearing placebo	6 months	200 (data analyse van 176)	the Hong Kong List Learning Test= Tests of learning and memory (assesses rate of learning, rate of forgetting, encoding and retrieval deficits, and learning strategies) & Rey- Osterrieth Complex Figure Test and Wechsler Memory Scale-Revised = visuospatial constructional ability and visual memory	°		yes	(47)
**23**	Huhn S	2018	Neuroimage	healthy adults	60-78	resveratrol	200 mg/day of resveratrol and 320 mg of quercetin	placebo (identical w/o resveratrol)	26 weeks	60 (53 over)	California Verbal Learning Task (CVLT, main outcome), the ModBent task,	°		yes	(48)
**24**	Jackson PA	2016	Nutrients	healthy adults, SMI	55-65	*ginkgo biloba*	Ginkgo biloba (240 mg)	2.24 g high oleic acid sunflower oil and 120 mg fish oil (32 mg DHA + EPA)	24 weeks	248 (84 completed)	Cognitive Demand Battery(CDB), Rapid Visual Information Processing(RVIP)	°		yes	(49)
**25**	Kanowski S	1996	Pharmacopsychiatr y	AD or multi-infarct dementia	>55	*ginkgo biloba*	240 mg EGb 761®, 22– 27% Ginkgo flavonoids, 2.8–3.4% ginkgolides A, B, C, 2.6–3.2% bilobalide	placebo (unclear)	24 weeks	216(156 /205 analysed)	CGI, SKT, NAB	+	WM	no (no raw measures available)	(50)
**26**	Kaschel R	2011	Phytomedicine	healthy adults	45–65	*ginkgo biloba*	240 mg EGb 761®, 22– 27% Ginkgo flavonoids, 2.8–3.4% ginkgolides A, B, C, 2.6–3.2% bilobalide	unclear, placebo tablets	6 weeks	188 (177 for Memory tasks)	standardised free recall paradigm, standardised recognition test	+	EM	yes	(51)
**27**	Kean RJ	2015	Am J Clin Nutr	healthy adults	60-81	*flavonoids*	549 mg hesperidin/L, 60 mg narirutin/L(250 mL twice per day)	low flavone drink	8 weeks	35-28 (cross-over)	Spatial Working Memory	+	EM	yes	(52)
**28**	Kent K	2017	Eur J Nutr	mild-moderate AD	>70	*anthocyanins*	200 ml/day of cherry juice	control juice with negligible amounts of anthocyanins	12 weeks	49	battery of seven cognitive tests: RAVLT, SOPT, Boston naming test, TMT(A and B), digit span backwards task, category verbal fluency and letter verbal fluency	+	EM	yes	(13)
**29**	Kreijkamp- Kaspers S	2004	JAMA	postmenopausal women	60-75	*isoflavones*	25.6 g/day of soy protein (containing 99 mg of isoflavones: 52 mg genistein, 41 mg daidzein, and 6 mg glycetein)	milk protein	12 months	202 (175 completed)	Rey auditory verbal learning test, measures of short- term and long-term verbal and visual memory, Doors test, Digit span test	°		yes	(53)
**30**	Kritz-Silverstein D	2003	Menopause	healthy adults	55-74	*isoflavones*	110 mg/day total isoflavones	identical placebo	6 months	56 (53 completed)	WMS Logical Memory and Recall	°		yes	(54)
**31**	Kuszewski JC	2020	J Nutr	healthy adults	50-80	*curcuminoids*	160mg/day curcumin or 160mg/day combined with fish oil supplementation (2000 mg/d DHA + 400 mg/day EPA)	fish oil supplementation (2000 mg/d DHA + 400 mg/day EPA)	16 weeks	152(134 completed, 126 analysed)	neuropsychological test battery	°		no (no raw measures available)	(55)
**32**	Le Bars PL	1997	JAMA	dementia	>45	*ginkgo biloba*	120 mg ginkgo	placebo (not specified)	52 weeks	309 (202 completed)	ADAS-cog	+	WM	no (no raw measures available)	(56)
**33**	Lewis JE	2014	BMC Complement Altern Med	healthy adults	>60	*ginkgo biloba*	120mg ginkgo leaf, 80mg whole gingko +others, +either 700mg choline vs OR anthocyanins mixture, green tea, vitamins etc.	cellulose, lactose, beet powder	6 months	97 (33+31+33)	TMT, Stroop	+	WM	no (no raw measures available)	(57)
**34**	Li S	2019	J Int Med Res	VaMCI	mean age 65	*ginkgo biloba*	3x19.2mg ginkgo	3x1.8mg pushen (traditional Chinese medicine)	12 weeks	64 (57 completed)	MoCA	°		yes	(58)
**35**	Mastroiacovo D	2015	Am J Clin Nutr	cognitively intact adults	61-85	*cocoa flavanols*	drink containing 993 mg/ day [high flavanol (HF)], 520 mg [intermediate flavanol (IF)]	48 mg [low flavanol (LF)] cocoa flavanols (CFs)	8 weeks	90	Verbal fluency test	+	WM	yes	(59)
**36**	Mazza M	2006	Eur J Neurol	mild-moderate AD	50-80	*ginkgo biloba*	daily: Ginkgo biloba: 160 mg	cholinesterase inhibitor: donepezil (5 mg daily dose), placebo	24 weeks	74 (60 completed)	SKT - Syndrome Kurz test (psychometric test battery for assessment of memory and attention), MMSE, CGI	+	WM	no (unspecific outcome)	(60)
**37**	McCarney R	2008	Int J Geriatr Psychiatry	dementia	>50	*ginkgo biloba*	120 mg daily (60mg twice a day); placebo: lactose-based	lactose-based placebo	6 months	176 (176 analysed)	ADAS-cog	°		no (unspecific outcome)	(61)
**38**	McNamara RK	2018	Neurobiol Aging	SMI (mild)	62-80	*anthocyanins*	berry powder 25g dry weight, or combined fish oil + blueberry	placebo isocaloric carbohydrates or fish oil	24 weeks	94 (21,24,26,23; 76 analysed)	TMT, verbal learning, fluency	°		no (no raw measures available)	(62)
**39**	Mix JA	2002	Hum Psychopharmacol	cognitively intact adults	>60	*ginkgo biloba*	180 mg/day of Ginkgo biloba extract	placebo (unclear)	6 weeks	262 (149 analysed)	WMS-R LM	+	EM	yes	(63)
**40**	Napryeyenko O	2007	Arzneimittelforschu ng	AD or VaD	>50	*ginkgo biloba*	daily: 2 × 120 mg EGb 761	unclear	22 weeks	395	SKT test battery	+	WM	no (no raw measures available)	(64)
**41**	Nasab NM	2012	J Pak Med Assoc	mild-moderate AD	50-75	*ginkgo biloba*	120mg ginkgo	4,5mg rivastigmine (cholinesterase inhibitor)	24 weeks	56(51 completed)	7minutes test, MMSE	-	WM	yes	(65)
**42**	Nilsson A	2017	PLoS One	healthy adults	50-70	anthocyanins	3x200ml berrymix (1325mg/l=811 mg/d)	placebo (matched to ingredients, pH etc) less fibre in the control drink	5 weeks	46(40 analysed, crossover)	dedicated sentence list - WM test	°		yes	(66)
**43**	Ochiai R	2019	J Alzheimers Dis	MCI	60-84	*chlorogenic acid*	2x553.6mg CGA/d	placebo (identical wo CDGs)l	12 weeks	34(28 analysed), crossover	ADAScog, TMT	+	WM	yes	(67)
**44**	Pase MP	2013	J Psychopharmacol	healthy adults	40-65	*cocoa flavanols*	500mg + 250 mg cocoa flavanols	protein, sugar, fat- matched; all had caffeine	30 days	87(77 analysed)	CDR battery	°		yes	(68)
**45**	Pipingas A	2008	Phytother Res	cognitively intact adults	50-65	*flavonoids*	960 mg of Enzogenol® and 120 mg of vitamin C. Enzogenol® is an aqueous extract from the bark of New Zealand grown Pinus radiata trees containing approximately 80% total proanthocyanins and other water-soluble flavonoids, flavonoid- conjugates and phenolic acids. Rich in proanthocyanins and contain a range of flavonoids including catechin, epicatechin, quercetin, dihydroquercetin, taxifolin and phenolic acids	vitamin c only	5 weeks	42	computer-based cognitive tasks for spatial working memory	+	WM	yes	(69)
**46**	Rainey-Smith SR	2016	Br J Nutr	healthy adults	40-90	*curcuminoids*	1500 mg/d total (1 × 500mg BCM-95®CG (BiocurcumaxTM) capsule three times a day):	placebo (unclear)	12 months	160	sixteen-item self- report Prospective and Retrospective Memory Questionnaire; Rey Auditory Verbal Learning Test; Subtests of Cogstate battery	°		yes	(70)
**47**	Ryan J	2008	J Psychopharmacol	healthy adults	60-85	*flavonoids*	3x50mg PYC	placebo (same size and appearance), unknown	3 months	120(101 completed)	CDR battery	+	WM	yes	(71)
**48**	Santos RF	2003	Pharmacopsychiatry	healthy men	60-70	*ginkgo biloba*	80mg extract (24%flavonoids)	placebo (same size and appearance), unknown	8 months	48 (all completers)	test battery	+	WM	yes	(72)
**49**	Schneider LS	2005	Curr Alzheimer Res	mild-moderate AD	>60	*ginkgo biloba*	1x120mg ginkgo or 2x120mg (240mg) ginkgo	placebo	26 weeks	513 (410 completed)	ADAS Cog	°		no (no raw measures available)	(73)
**50**	Schneider LS	2019	Menopause	perimenopausal women	45-60	*isoflavones*	50mg+100mg/d phytoserm (=isoflavones)	placebo (same size and appearance), unknown	12 weeks	71 (66 completed)	composite measures	°		no (no raw measures available)	(74)
**51**	Siddarth P	2020	Am J Clin Nutr	normal or MCI	50-75	*anthocyanins*	236,5ml pomegranate/d (368mg punicalagins, ca 100 anthocyanins, etc	placebo (same taste, sugar etc)	12 months	261(200 completed)	Brief Visuospatial Memory Test-Revised (BVMT-R) and Buschke Selective Reminding Test (SRT)	+	EM	yes	(75)
**52**	Small BJ	2014	Rejuvenation Res	cognitively intact adults	65–85	*anthocyanins*	900 mg NT-020 PlusBiovin / day (containing green tea extract (95% polyphenols), VitaBlue (40% polyphenols, 12.5% anthocyanins from blueberries), grape polyphenols (including 5% resveratrol), carnosine, vitamin D3 (2000 UI/serving) and Biotin 40 mg.	placebo (unclear)	2 months	113	Auditory Verbal Learning Test (AVLT; immediate and delayed recall) voor episodic memory;	°		yes	(76)
**53**	Snitz BE	2009	JAMA	healthy elderly, MCI	72-96	*ginkgo biloba*	2x120mg ginkgo	placebo (unknown)	6,1 years	3072(1545 +1542 completed)	Stroop, TMT and other	°		yes	(77)
**54**	Solomon PR	2002	JAMA	healthy adults	60-82	*ginkgo biloba*	3x40mg ginkgo/day	gelatine capsules	6 weeks	230 (203 completed)	Digit span, Stroop and other	°		yes	(78)
**55**	Suominen MH	2020	Exp Gerontol	healthy adults	65-75	*cocoa flavanols*	50g dark chocolate (410mg flavanols/day)	50g dark chocolate (86mg flavanols/ day)	8 weeks	104 (100 completed)	TMT	°		yes	(79)
**56**	Thaung Zaw JJ	2021	Clin Nutr	postmenopausal women	45-85	*resveratrol*	75mg trans-resveratrol	several inert excipients	14 weeks	146 (110 analysed, cross-over)	TMT, list sorting, wordlist, picture memory	+	WM, EM	yes	(80)
**57**	van Dongen M	2003	J Clin Epidemiol	mild-moderate AD/ VaD or age-associated cognitive impairment	>50	*ginkgo biloba*	240 mg/day ginkgo biloba special extract (high dose) or 160 (usual dose) mg/day	placebo (unclear)	24 weeks	214	NAI-ZN-G; self-perceived health and memory status	°		yes	(81)
**58**	Whyte AR	2018	Nutrients	healthy adults	65-80	*anthocyanins*	450mg/day blueberry powder (35mg polyphenols, 1.35mg anthocyanins)or 900mg blueberry powder (70mg polyphenols, 2.7mg anthocyanins) or 100mg blueberry extract (50mg polyphenols, 7mg anthocyanins)	maltodextrin	6 months	122 (112 completed)	AVLT, object recognition, serial substractions, Stroop	°		no (no raw measures available)	(82)
**59**	Wightman EL	2018	Nutrients	healthy adults	50-70	*flavonoids*	475mg or 950 mg/day *Sideritis scardica* (Greek Mountain Tea) or 240mg ginkgo/day	maltodextrin	28 days	155 (140 completed)	own tests, global scores available	+	WM	yes	(83)
**60**	Witte AV	2014	J Neurosci	healthy overweight adults	50-80	*resveratrol*	200 mg/day of resveratrol and 320 mg of quercetin	placebo (corn oil)	26 weeks	46	Auditory Verbal Learning Test (AVLT)	+	EM	yes	(84)
**61**	Wong RH	2013	J Hypertens	obese healthy men and postmenopausal women	40-75	*resveratrol*	75mg trans-resveratrol	placebo (identical w/o resveratrol)	6 weeks	28 (cross-over)	Stroop	°		yes	(85)
**62**	Woo J	2003	Menopause	postmenopausal women	50-65	*isoflavones*	100mg isoflavones	hormone replacement (estrogen/ progesterone) OR no treatment	3 months	127 (completers)	TMT, word list learning	°		yes	(86)
**63**	Yakoot M	2013	Clin Interv Aging	MCI	50-80	*ginkgo biloba*	G. biloba leaf extract 120 mg (standardized to contain 24% flavonoid glycosides and 6% terpenoids) and 150 mg of P. ginseng alcohol root extract (containing 40%– 80% ginsenosides).	750 mg of natural lyophilized royal jelly (standardized to at least 6% of 10-hydroxy-2decenoic acid)	4 weeks	66 (60 completed)	MMSE	+	WM	no (unspecific outcome)	(87)
**64**	Yancheva S	2009	Aging Ment Health	probable AD w neuropsychiatric symptoms	>50	*ginkgo biloba*	2x120mg (240mg/day ginkgo) or combined with donezepil 5-10mg/ day	5-10mg donezepil	22 weeks	96 (88 completed)	SKT	°		no (no raw measures available)	(88)
**65**	You YX	2021	Nutrients	MCI	60-75	*flavonoids*	2x250mg CC +250maltodextrin	2x500mg maltodextrin	12 weeks	48 (47 completed)	digit span, AVLT	+	WM, EM	yes	(89)
**66**	Zhang SJ	2012	Asian Pac J Trop Med	cerebral infarction patients/vascular cognitive impairment	60-75	*ginkgo biloba*	3x75mg aspirin/day + 3x40mg ginkgo	3x75mg aspirin/day	3 months	80	MOCA	+	WM, EM	yes	(90)

### Evaluation of Effect Sizes

Effect size d for all studies with available data [mean, standard deviation (SD) or *F*-statistics] was computed in the following way: (1) for studies with available mean and SD for 1 timepoint for 2 groups, we calculated effect sizes using the “Means, Standard Deviations, and Sample Sizes” algorithm available at https://www.campbellcollaboration.org/escalc/html/EffectSizeCalculator-SMD1.php, (2) for studies with mean and SD for 2 timepoints for 2 groups (repeated measure design), we calculated effect sizes for mean differences of groups with (un)equal sample size within a pre-post-control design using the algorithm at https://www.psychometrica.de/effect_size.html, and (3) for studies with *F*-statistics only (no mean, SD available), we calculated effect size according to the algorithm at https://www.campbellcollaboration.org/escalc/html/EffectSizeCalculator-SMD4.php. If necessary, standard error (SE) was transformed to SD by calculating SD = SE × sqr (*n*).

Effect size error SE_d_ was computed according to:


$$\begin{array}{l} SE_{d}=\;\sqrt{\frac{n_{1}+n_{2}}{n_{1}n_{2}}+\;\frac{d^{2}}{2\left(n_{1}+n_{2}\right)}} \end{array}$$

with effect size (d), intervention sample (n_1_) and control sample (n_2_). For those memory outcomes, where higher scores relate to lower performance (i.e., reaction time), effect size valence was inversed, to represent memory improvements. At maximum four outcome measures (2 WM, 2 EM) were included in the meta-analysis per study due to feasibility. Effect sizes were calculated for the most sensitive measures (for WM e.g., Stroop inference reaction time and for EM e.g., delayed recall of a 10–15 words list) and in case of more than two groups or timepoints, for the group that had a single formula and/or the highest dose compared to placebo/lowest dose; and for the longest intervention period. A deviation of this rule was made for Wightman et al. (83) reporting effect sizes for (1) *Sideritis scardica* extract and (2) *Ginkgo biloba* compared to placebo.

To analyse overall effect sizes of the included studies and a potential positivity bias in the publication records, we compared effect sizes of WM and EM outcomes, respectively, with forest and funnel plots in the software JASP developed and distributed by JASP Team (91), JASP (Version 0.14.1) [Computer software]. Inference of a random effects meta-analysis per memory category (EM and WM, respectively) was computed with the restricted maximum likelihood methods by pooling all polyphenol interventions and subsequently with sub-analyses in subgroups.

## Results

### Study Characteristics

The included 66 studies had study durations varying between 4 weeks and 2.5 years and sample sizes between 42 and 350 participants. The investigated polyphenol extracts were administered as tablets, capsules, powder, chocolate bar or drink and comprised a large variety of polyphenol molecules. According to Truzzi et al. (92), polyphenols can be classified in four subclasses according to the number of inert phenolic rings, i.e., flavonoids, phenolic acids, stilbenes and lignans; others that do not fall into those categories are polyphenols such as e.g., curcuminoids. To compare the studies included in this review, we decided to group studies according to the examined extract, polyphenol substance or polyphenol subclass that the authors described as main or most effective ingredient. This resulted in the following subcategories: *Ginkgo biloba* leaf extract (26 studies), soy isoflavones extracts (11 studies), anthocyanins (eight studies, e.g., blueberry extract, pomegranate, cherry juice, etc.), cocoa flavanols (four studies), flavanoid extracts (five studies, e.g., *Sideritis scardica, Pinus radiata* bark, etc.), chlorogenic acids (two studies), curcuminoids (four studies), and resveratrol (five studies). Note that all subcategories except chlorogenic acids, curcuminoids, and resveratrol belong to the same subclass of flavonoids (92), and that most extracts contained multiple polyphenols, leading to a certain overlap between study subgroups.

The majority of studies reported to use a placebo tablet, capsule, powder or drink as a control condition for supplementary polyphenols. These placebos comprised for example the intervention formula but only with negligible or no amounts of polyphenols, for example in tablets, capsules or drinks. Some studies incorporated isocaloric maltodextrin or cow milk protein (i.e., for soy isoflavone) as placebo, or studied the effects of polyphenols alone, or added to a certain treatment, e.g., fish oil or vitamins or aspirin, compared to those treatments alone, sometimes including a third arm without any treatment/placebo. Other studies, in some cases including 3–4 study arms, tested effects of polyphenols vs. medications, such as rivastigmin or donezepil in the case of cognitive decline, or hormone replacement therapy in the case of peri/postmenopausal women, or in comparison to lower doses of the polyphenol, for example flavanols. For more information about the study characteristics, (see Table 1).

### Adverse Events

Most, but not all studies reported on adverse events. Some studies did this extensively, others scantly reported on adverse events. Overall, polyphenols were well-tolerated. Most often, when adverse events were reported, the number and type of adverse events did not differ significantly from the placebo group. One study on *Ginkgo biloba* effects reported the occurrence of stroke in one participant, which could not equivocally related to the treatment (38). Recurring complaints in the reviewed studies were gastrointestinal complaints.

### Reported Effects of Polyphenols on Memory

Overall, in this systematic review, 32 out of 66 studies (48.5%) found at least one significant improvement on any of the memory outcomes measured, while 31 (47%) did not report significant performance, and 3 (4.5%) did report decreases in memory in at least one measure (Table 1). However, these 66 studies analyzed a multitude of different outcome measures and different polyphenol substances or mixtures. All studies included at least one WM test outcomes, and the majority did also report EM outcomes. However, studies including dementia patients most often included only global assessments with relatively coarse measures of memory performance, such as the ADAS-Cog or SKIT, due to the severity of disease. This somewhat limits the interpretability of possible polyphenol effects on episodic memory in patients suffering more severe cognitive decline.

Tests assessing WM usually ask participants to remember information for a short amount of time and to manipulate this information. Examples of tests that measure WM are the digit span task, where participants have to repeat a series of numbers in the same or reversed order, or the serial subtraction task, where participants are assigned to keep on subtracting the number three or seven from a random number between 800 and 999 (18, 53). Another frequent test was the trail making test (TMT) B, which gives a working memory estimate of mental flexibility. Assessing EM typically entails the visual or verbal presentation of information to a participant and later measures whether this information is remembered (22). An example of an EM test is the Rey Auditory Verbal Learning Test (RAVLT), where participants hear a list of 15 words spoken out loud and are asked to recall the words immediately and after a delay and are asked what words they recognized from a 30-words list comprising old, new and distractor words (53). Forty-five studies included healthy older adults, with some focusing on overweight/obese participants and 12 included only peri/postmenopausal women, one only men. Eleven studies included subjective (SMI) or minor neurocognitive impairment (MCI), while another 14 studies included dementia of the Alzheimer's type (AD) or vascular dementia (VaD), or both. Depending on population, most studies had a relatively broad age range older than 40, 45, or 50, some focused on 65–80 years old only.

### Effects of Polyphenols on Working and Episdoic Memory (WM/EM)

#### Ginkgo biloba

Almost half of the studies included in the review, i.e., 26, investigated the effects of *Ginkgo biloba*, in both healthy and disease samples, two with ginseng added to the treatment. *Ginkgo biloba* leaf extract is comprised of different polyphenols but particularly—similar to soy or certain other plant extracts—rich in flavonoids. The duration of treatment lasted from 4 weeks to up to 3.5 years. Fourteen of those studies reported at least one significant improvement, 11 no changes and two a worsening of functions. Notably, two of the longest studies over 3.5 and 6.5 years, respectively, reported diverging results: the first in 202 completers found a significant beneficial effect of 120 mg/day ginkgo tablets compared to placebo tablets in the ADAS-cog test and the GEFRI test (*p* = 0.04) in dementia patients (56), while the second longer one in a very large group (*n* = 3,072 completers) and after a doubled dosage (240 mg *Ginkgo biloba*) could not detect significant differences in healthy older or MCI patients (77). Due to the large sample size and long duration, the latter study is of particular interest, as well as the first one, however we could not include it in the meta-analysis based on missing specific memory outcomes (56). Two shorter studies in mild to moderate AD with doubled dosage over 6 months compared to placebo in around 400 participants also reported benefits (46, 64). However, other large trials with 410/214 completers reported no significant differences for improvements after 6 months in the ADAS cog test (73) (Table 1). Some studies also included subjective measures of memory performance, which is by some regarded as biomarker of later cognitive function (“subjective memory impairment”), however a less objective measure of current memory performance and thus not further considered in this review. Notably, two studies reported a worsening of function compared to control condition, one in healthy adults after 160 mg/day in 78 completers (however for EM outcome), and another one in patients with mild to moderate AD when comparing 120 mg/day ginkgo to 4.5 mg/day rivastigmin treatment (a cholinesterase inhibitor).

#### Soy Isoflavones

Isoflavones show similarities with steroidal estrogens and are suggested to counteract menopause-related estrogen deficiency possibly causing cognitive decline (24, 47). Soy isoflavones were examined in nine studies in peri- or postmenopausal women, one study included also men and another one AD patients. Overall, most studies (72%) could not detect any significant effects of isoflavones (mostly from soy) in dosages between 35 and 160 mg/day over 3–12 months, including a relatively large study with 175 women over the course of 1 year (53). One study in 79 women over three groups reported a worsening of a verbal working memory outcome after 16 weeks of soy isoflavone intake as soymilk, compared to cow milk or isoflavone supplement intervention (40). A cross-over study of 6 months isoflavone tablets compared to placebo (*n* = 76), however, as well as larger (*n* = 313), longer term intervention of soy isoflavones against milk protein-based placebo over 2.5 years, showed improvements in a working memory outcome [TMTB (34)] and in episodic memory (24), respectively. It could be speculated that isoflavones are effective in earlier age only because of their possible action as estrogen-related effects. Some studies indeed showed that effects are mainly found several years before menopause (i.e., perimenopause), when the ovaries start to produce less estrogen, or in young postmenopausal women (93, 94). However, two studies in perimenopausal women included in this review do not support this claim (41).

#### Berry, Cherry, Grape and Tea Extracts, Curcuminoids, and Related Supplements

Berry, cherry, grape, and green tea extracts are among other polyphenols rich in anthocyanins, evaluated in nine studies. Other extracts included cocoa flavanols (studied in four studies), flavonones (one study), curcuminoids (four studies), and rosmarinic acids (one study). Overall, study results were mixed with nine studies reporting no significant effects and nine studies reporting significant outcomes for WM or EM tasks. One study reported improvements in spatial and WM, but not EM, after intake of rosmarinic acid, containing spearmint extract for 90 days compared to identical placebo supplement (0 mg spearmint), after three months in a group of 87 healthy older adults (45, 69). Considering anthocyanins, for example administered as grape juice or blueberry extracts, most studies including healthy participants could not detect significant changes over 5 weeks to 6 months (four studies). For example, one study performed in 113 healthy elderly did not find any significant improvements on WM or EM outcome measures between the experimental and control group after 2 months (76). Study dosages were partly unclear, as for example, Whyte et al. (82) administered between 35 and 70 mg polyphenols per day and Small et al. (76) reported administering 900 mg of the supplement NT-020 per day, but it was unclear what the amount of which polyphenol was per dosage. In a relatively large sample (*n* = 122) of elderly with subjective self-reported memory complaints (82), the group receiving low dose polyphenols per day, including 7 mg anthocyanin per day, showed a significant improvement compared to placebo on one EM outcome measure after 3 months of supplementation, but not after 6 months. No significant improvements were found on the other five outcome measures of the verbal learning task or on a visual EM task.

In a population of 49 older adults with mild-to-moderate dementia and 138 mg anthocyanins per day intervention, however, Kent et al. (13) found significant improvements on all three measures of EM after 3 months, with moderate to large effect sizes (13). Bensalem et al. (31) reported dosing 258 mg flavonoids per day and showed a significant improvement in one of the three EM outcome measures after 6 months in a sample of 215 healthy elderly (31), and a secondary analysis revealed that polyphenol supplementation significantly improved all three measures of EM in a subgroup with the highest cognitive decline.

The influence of flavanols derived from cocoa was measured in both cognitively intact elderly and elderly with mild neurocognitive disorder (37, 59). In two studies, results showed that participants drinking high and medium amounts of flavanols performed significantly better on a WM task than participants drinking low amounts of flavanols. In Mastroiacovo et al. (59), the high and medium flavanol groups reduced the time to finish a WM task over 8 weeks with 17 s (21%) and 14 s (18%) respectively, compared to 1 s (1%) in the low flavanol group. However, two studies in 77–100 healthy adults could not detect significant effects after 4–8 weeks intervention with daily drinks or chocolate bars against a protein shake or a low-dose flavanol chocolate, respectively, as control (68, 79). A similar impression comes from four studies on curcuminoids, with two showing no significant results in 134–160 healthy olders after 16 weeks-−12 months intervention (55, 70) and two studies from the same group in 60–79 participants reporting significant improvements in working memory measures after 4 and 12 weeks (18, 36). Here, it seems as if a shorter study duration (i.e., 4 weeks) more likely resulted in positive results, while an intervention period of 1 year did not lead to a difference in composite scores between the placebo and intervention group. On the other hand, the power of the studies with insignificant results was bigger, as the sample size were larger. The dosages of those studies are difficult to compare. Cox et al. (18) for example administered 80 mg curcumin per day. However, Rainey-Smith et al. (70) reported administering 1,320 mg curcuminoids but not mentioning the precise amount of other polyphenols.

In sum, the evidence for a beneficial effect on EM or WM on anthocyanins, flavanols and curcuminoids, is still limited, with a tendency toward more positive effects when cognitive decline is more severe based on two studies only. In line with this, though, the sample of Small et al. (76), showing no significant differences at all, was highly educated and therefore a ceiling effect of the memory task cannot be ruled out. Note also a lack of homogeneity with regard to dosages of different polyphenol subclasses, rendering a direct comparison of study results difficult: some of the flavanol studies for instance varied between 990 and 520 mg flavanol per day, while in the isoflavone studies, lower but still varying doses of 35–160 mg of isoflavones were administered.

#### Stilbenes

Supplementary doses of isolated polyphenols were rare and often combined with others derivatives or substances, such as in studies examining resveratrol, a polyphenol of the stilbene subclass (five studies). These studies tested postmenopausal women and healthy older adults with again mixed results. For example, Evans et al. (39) administered 150 mg resveratrol per day for over 14 weeks to 80 participants, while Huhn et al. (48) administered 200 mg per day plus 320 mg quercetin for over 26 weeks to 60 participants. Considering WM, although Huhn et al. (48) found no significant improvements, pattern recognition on a spatial WM-related test decreased in the placebo group and did not change in the resveratrol group. This result could suggest that resveratrol helps to preserve cognitive function, while memory otherwise subtly declines with aging. However, this effect was not seen in Evans et al. (39), where the performance in both the placebo and the resveratrol group increased with about the same amount. While quercetin was given to increase bioavailability of resveratrol, it cannot be excluded that a positive result related to the flavonoid-related benefits of quercetin, administered simultaneously in Huhn et al. (48). Considering EM, significant improvements were found in a sample of 46 healthy older adults and in a sample of 80 postmenopausal women (39, 84). Witte et al. (84) administered 200 mg resveratrol and 320 mg quercetin for 26 weeks. In both studies, the positive effect was only found on delayed recall and not on immediate recall of a verbal learning task. According to Evans et al. (39), the effect size was small. The above mentioned study by Huhn et al. (48) researching 60 healthy elderly could not confirm these findings, as no significant improvements in delayed recall, nor on three other outcome measures of a verbal learning task were found after 26 weeks (48). Again, it is imported to highlight that both Witte et al. (84) and Huhn et al. (48) also administered quercetin, to increase the bioavailability of resveratrol, so the results of these studies might be a combined effect of flavonoids and stilbenes.

Related, while one study did not report significant differences after resveratrol in obese older adults (85), others studied an overweight population and found significant improvement in EM performance after resveratrol administration (84). Here, the increased EM performance was associated with improvements in glucose metabolism (i.e., lower HbA1c levels). In addition, ameliorated memory performance was associated with increased functional connectivity between the hippocampus and the medial prefrontal cortex, suggesting a mechanistic link. In addition, a study with cocoa flavanol drink observed significant reductions in blood insulin after treatment, which also explained variance in the memory increases after treatment (55), again pointing to glucose metabolism as a potential mechanism. Positive verbal memory results for 75 mg/day trans-resveratrol intake compared to placebo capsules were also found in postmenopausal women in a very recent study after 14 weeks each in a cross-over design, including 110 women (80).

#### Evaluation of Effect Size

Considering the difference between the reviewed studies, overall, results appeared to be independent of sample size and study duration. A substantially larger sample size, leading to a greater power to detect an effect that is present, did not always lead to significant results in some samples, similar to smaller samples, suggesting that the effect of polyphenols on memory depends on other factors.

**Figure 2:**
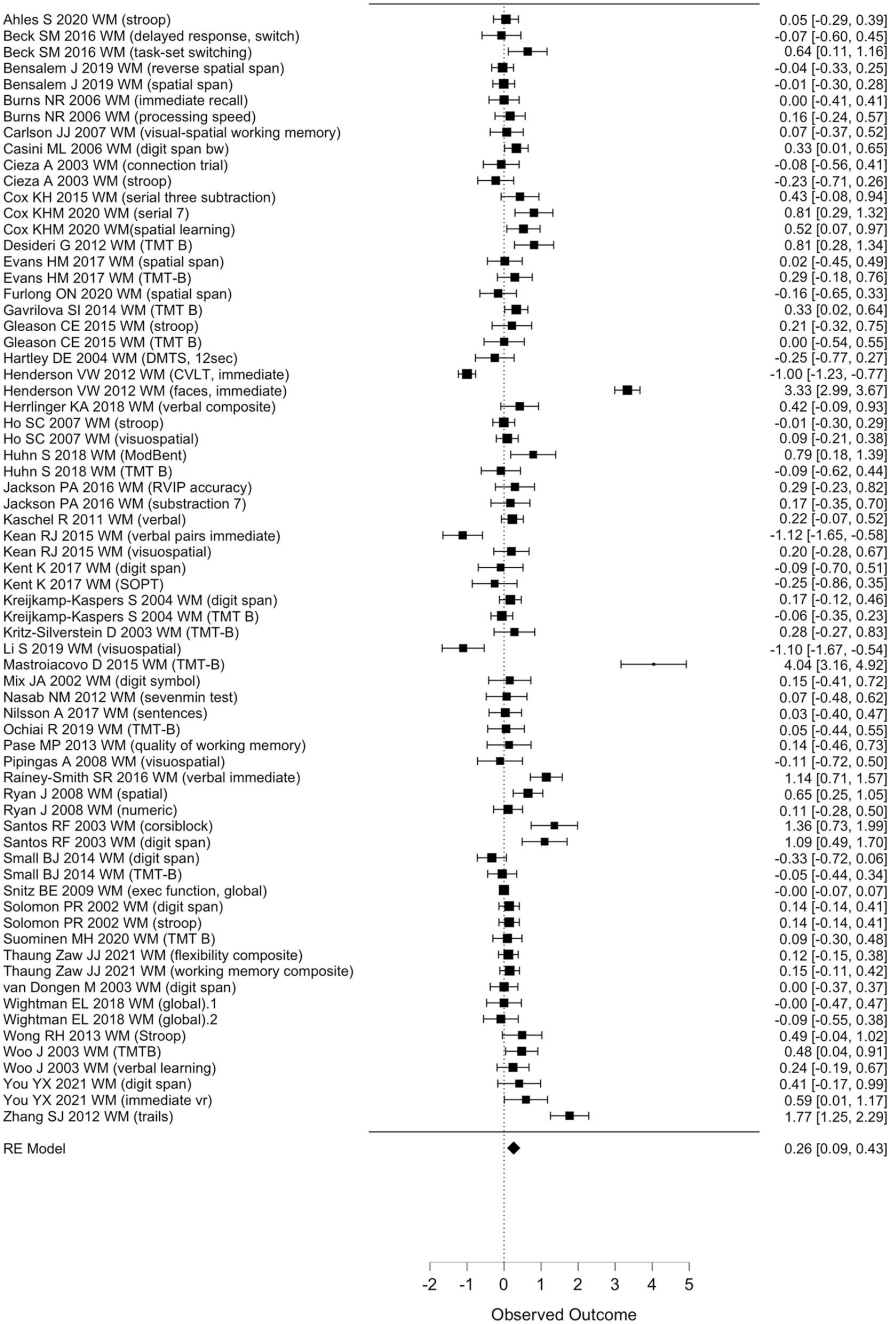
Forest plot for observed effect sizes (d) and standard errors (SE) for available study results for WM. RE, regression estimate.

Taken together, available effect sizes of WM outcomes after polyphenol intake interventions appeared in the majority either small or non-existent, with the exception of some studies in both directions and two studies reporting extremely large positive effects on immediate face recognition (24) and TMT-B (59) (Figure 2).

**Figure 3:**
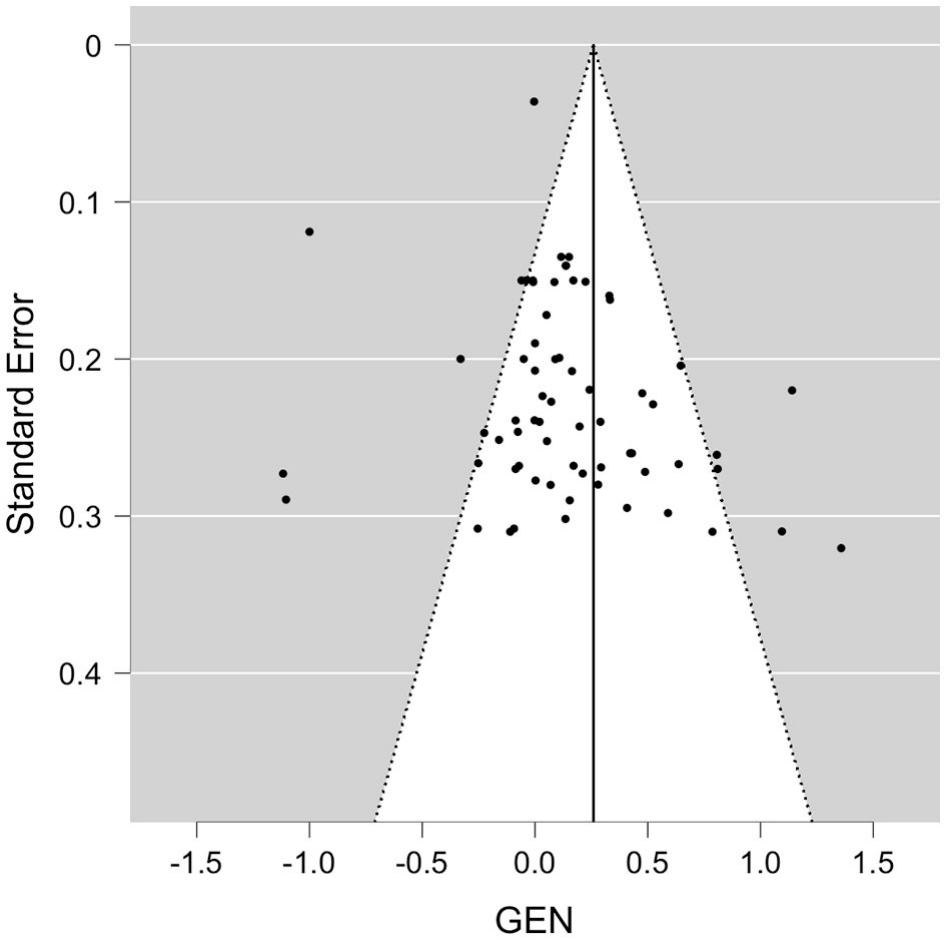
Funnel plot for available study results for WM. GEN, general effect result per study.

When pooled in a meta-analysis, polyphenols showed a small overall positive effect on working memory [Wald test, *b* = 0.26 (95% CI: 0.09; 0.43), *z* = 3.07, *p* = 0.002]. The between-study heterogeneity variance was estimated at τ^2^ = 0.44 (95% CI: 0.32–0.7), with an *I*^2^ value of 93% (95% CI: 91–96%), indicating that statistical heterogeneity between studies was highly significantly large [*Q*_(66)_ = 710, *p* < 0.001]. The associated funnel plot indicated a positivity bias in the publication record, according to visual inspection and Egger's regression test (*p* = 0.017) and a trend in the rank correlation test (Kendall's τ = 0.15, *p* = 0.065) (Figure 3). To further evaluate whether the two relatively large RCTs (*n* = 90–313) that added large positive effect sizes to the meta-analysis had considerable influence on the results, we re-run the meta-analysis excluding these studies (24, 59), however, while the pooled effect size became somewhat smaller, significance level remained unchanged [*b* = 0.29, *z* = 3.6, *p* < 0.001 (95% CI: 0.08–0.28)], with not much lowered between-study heterogeneity [*I*^2^ = 77%, *Q*_(65)_ = 205, *p* < 0.001]. Note that a positivity bias could no longer be observed (Rank test and Egger's test, *p* > 0.18). When focusing on study outcomes that derived from comparisons of placebo formulas without polyphenols or no treatment as control condition (i.e., excluding study outcomes from comparison to lower dosages as control, or from polyphenols compared to fish oil, medications or to alternative extracts), overall results remained stable to the overall meta-analysis (59 outcomes, *b* = 0.26, *p* > 0.001, Rank test *p* = 0.045).

Studies including mainly healthy samples showed similarly a significant effect size on average, yet the likelihood of a positivity bias in those studies was increased (majority of studies, 55 outcomes), *p* < 0.032. In studies including mild cognitive impairment or dementia patients (14 outcomes), though, no positivity bias emerged, and effects were no longer significant (*b* = 0.2, *p* = 0.224). Duration of studies did not affect meta-analysis outcomes that much (<6 months: *b* = 0.19, *p* = 0.011; 6 months or longer: *b* = 0.47, *p* = 0.053), however Funnel asymmetry could not be excluded, and the majority of studies tested <6 months of intervention period. When restricting analyses for studies on ginkgo (23 outcomes, 17 studies, note also a large proportion of patient samples here), meta-analysis on effect sizes did not reach significance for a positive effect (*b* = 0.2, *p* = 0.075) and a positivity bias based on Funnel plot asymmetry was no longer observed (*p* > 0.35). Similarly, meta-analysis for isoflavones (12 outcomes, eight studies; *b* = 0.3, *p* = 0.28, Funnel asymmetry, *p* > 0.11), anthocyanins (eight outcomes, five studies; *b* = −0.06, *p* = 0.38, Funnel asymmetry *p* > 0.25) and flavonoids (eight outcomes, five studies; *b* = 0.1; *p* = 0.62, asymmetry *p* > 0.64) did not show significant effects. However, studies examining other extracts containing resveratrol (seven outcomes, four studies; *b* = 0.19, *p* = 0.013; asymmetry *p* > 0.22) supported a significant positive average effect size. Note that sample size in the subgroup-meta analyses was considerably reduced. No subanalysis was computed in groups with <5 outcomes.

**Figure 4:**
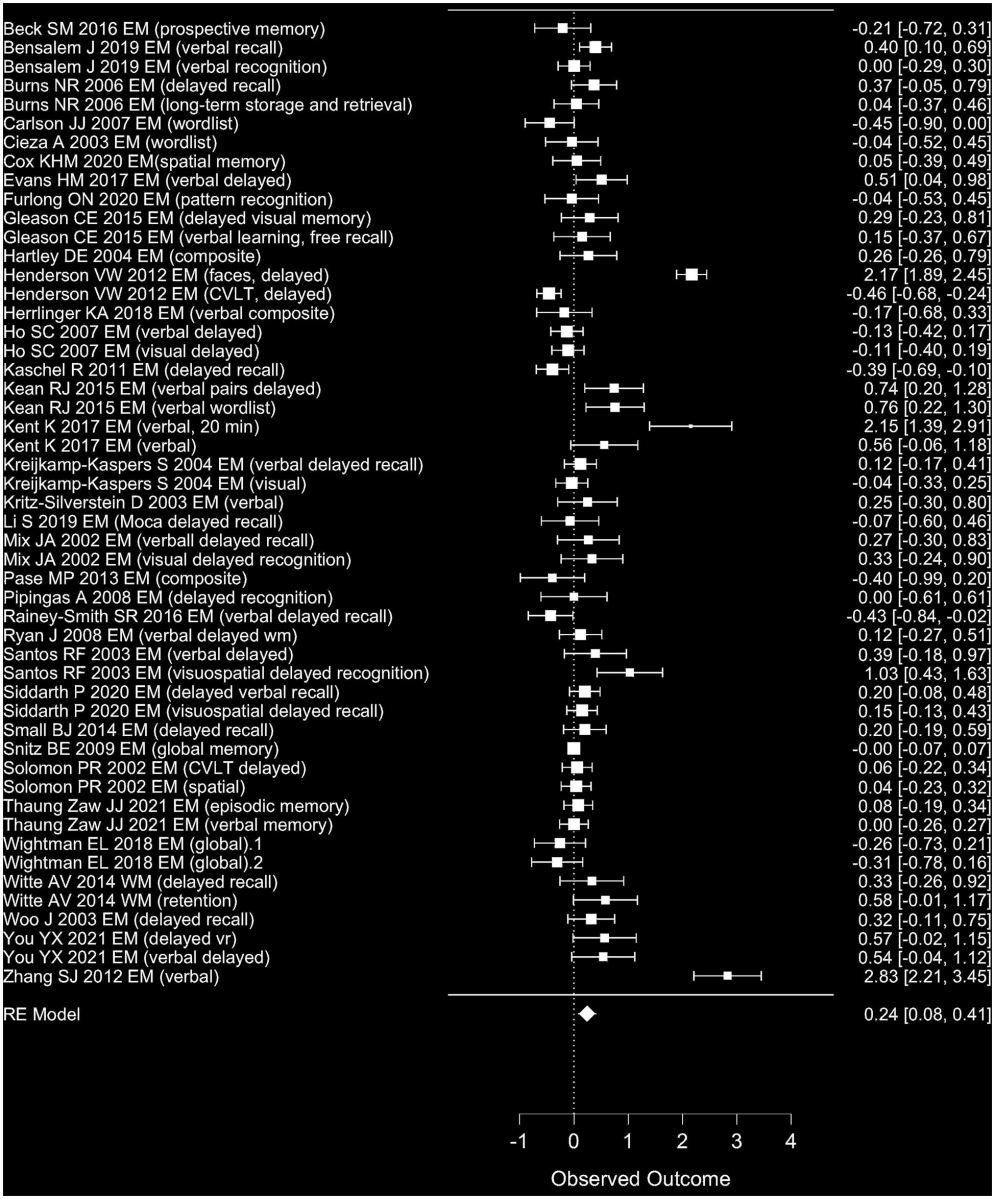
Forest plot for observed effect sizes (d) and standard errors (SE) for available study results for EM. RE, regression estimate.

**Figure 5:**
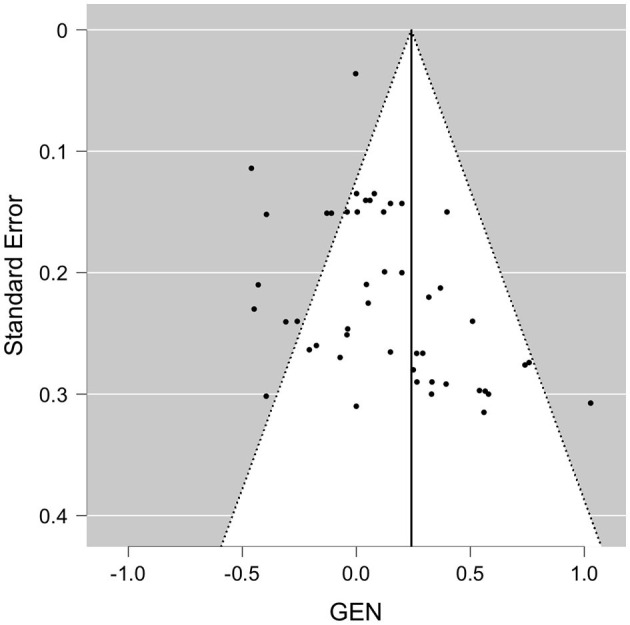
Funnel plot for available study results for EM. GEN, general effect result per study.

Evaluating EM, the included effect sizes indicated a small positive effect of polyphenol intake on EM outcomes on average (Figure 4). Accordingly, polyphenols showed a significant effect on episodic memory when pooled in a meta-analysis [Wald test, *b* = 0.24, *z* = 2.88, *p* = 0.004 (95% CI: 0.08–0.41)]. The between-study heterogeneity variance was moderate to large, estimated at τ^2^ = 0.31 (95% CI: 0.21–0.54), with an *I*^2^ value of 91% (95% CI: 88–95%), pointing toward a high statistical heterogeneity between studies [*Q*_(50)_ = 430, *p* < 0.001]. Notably, both the rank correlation test (Kendall's τ = 0.34, *p* < 0.001) and the Egger's regression test (*p* = 0.01) indicated significant asymmetry of the associated funnel plot. The funnel plot did not indicate according to visual inspection more strongly positive effect sizes had been reported in larger studies than could have been expected (Figure 5). Three small to large RCTs with small to longest durations added large effect sizes of positive sign (delayed recognition of faces, verbal recall) to the meta-analysis. When excluding these study (13, 24, 90), though, the pooled positive effect size became somewhat smaller yet remained statistically significant [*b* = 0.21, *z* = 2.39, *p* = 0.017 (95% CI: 0.02–0.18)], with a reduced between-study heterogeneity (*I*^2^ = 51%) yet still evidence for positivity bias (asymmetry, *p* > 0.021). When focusing on study outcomes that derived from comparisons of placebo formulas without polyphenols or no treatment as control condition (i.e., excluding study outcomes from comparison to lower dosages as control, or from polyphenols compared to fish oil, medications or to alternative extracts), overall results remained stable to the overall meta-analysis (47 outcomes, *b* = 0.23, *p* = 0.009, Rank test *p* < 0.001).

Note that a meta-analysis of those studies including mainly healthy samples did not reach statistical significance, yet the likelihood of a positivity bias in those studies was decreased majority of studies, 40 outcomes, *b* = 0.14, *p* = 0.069; Funnel asymmetry, *p* > 0.052. In studies including mild cognitive impairment or dementia patients (14 outcomes), effects were again significant, with a smaller estimate (*b* = 0.64, *p* = 0.019) and again positivity bias (Funnel asymmetry, *p* < 0.032). Duration of studies did not affect meta-analysis outcomes that much (<6 months: *b* = 0.23, *p* = 0.02; 6 months or longer: *b* = 0.25, *p* = 0.097), however Funnel asymmetry could not be excluded, and the majority of studies tested again <6 months of intervention period. When restricting analyses for studies on ginkgo (17 outcomes, 13 studies, note also a large proportion of patient samples here), meta-analysis on effect sizes did not reach significance for a positive effect (*b* = 0.22, *p* = 0.19) and a positivity bias based on Funnel plot asymmetry could still not be excluded (Rank test, *p* = 0.006). Similarly, meta-analysis for isoflavones (11 outcomes, seven studies; *b* = 0.23 *p* = 0.28, Funnel asymmetry, *p* > 0.24) did not show significant effects. In contrast, the meta-analysis for anthocyanins (seven outcomes, four studies; *b* = 0.45, *p* = 0.05, Funnel asymmetry Egger's test, *p* = 0.001), for other extracts containing flavonoids (seven outcomes, five studies; *b* = 0.34; *p* = 0.029 Funnel asymmetry *p* > 0.3) and trend-wise for resveratrol (five outcomes, three studies; *b* = 0.21; *p* = 0.067 asymmetry Egger's test, *p* = 0.024) somewhat indicated a positive average effect size, yet both groups raised awareness with regard to positivity bias. Note that sample size in the subgroup meta-analyses was considerably reduced. No subanalysis was computed in groups with <5 outcomes.

## Discussion

Based on this systematic review of 66 reported RCTs testing the effects of polyphenols on memory, 33 studies found a significant improvement on at least one memory outcome measure after polyphenol administration, while 30 did not find any significant effects, and three reported a worsening compared to control condition. Reporting was based on a variety of WM and EM outcome measures, of which a selection only were reported as significantly improved after polyphenol consumption compared to placebo with, if reported, small to large effect sizes. Considering the available and calculated effect sizes from available outcomes of 49 studies representing core WM and EM measures, pooled meta-analyses supported a small positive effect on both WM and EM with a mean effect size of 0.26 and 0.24, respectively. However, Funnel plot asymmetry tests detected a significant positivity bias for both WM and EM meta-analysis, questioning the validity of results. When excluding studies with very large positive effects, though, meta-analyses remained significant for a small effect. Our review further indicated a large heterogeneity between studies and outcomes studied, in terms of polyphenol formula and exact memory test used, study duration, sample size and characteristics, as well as considering statistical variance in effects sizes.

### Working and Episodic Memory

The evaluated studies can be summarized to show a small positive effect on both WM and EM with similar estimates, however, note that this finding needs to be interpreted with caution due the indicated positivity bias in reporting of polyphenol studies. Still, it has been argued that polyphenols might improve brain function. For example, Brickman et al. (95) showed that specific parts of the hippocampus are activated after polyphenol consumption. When high cocoa-flavanol consumption was compared to low consumption, the middle part (i.e., the body) of the hippocampus was activated. The activation was also associated with higher memory performance. It should be noted though, that this study was performed in a small sample of 37 participants divided into two groups. The hippocampus is necessary for accessing short-term memory representations during WM tasks (20, 96), and hippocampal and parahippocampal areas, parts of the medial temporal lobe (MTL), are critically involved in EM for encoding and retrieval (20, 96), whereas MTL lesions do not lead to extreme WM performance degradation. Later studies however indicated the involvement of the MTL in WM tasks, especially when associations (e.g., location and color) need to be made (96–98).

Future studies involving neuroimaging techniques could help to further understand the neurobiological mechanisms underlying potential benefits of polyphenols on WM and EM.

### Possible Sources of Study Heterogeneity or Bias

In this review, we did not observe strong systematic associations between memory improvement and sample size, study duration and mean age. Indeed, significant results were found in studies with long-term follow-up assessment after 2.5 years and in those with follow-up after 6 weeks. Additionally, bigger sample sizes, and thus greater power to detect an effect that is present did not result in more (in)significant findings. Also, the mean age of the samples did not systematically lead to significant or insignificant study results, however, some differences were observed regarding study population and polyphenol subclass.

### Polyphenol Subclasses and Dosage

Overall, results of the polyphenol subclasses were mixed. The percentage of significant memory outcome measures for *Ginkgo biloba*, other polyphenol-rich extracts, flavonoids, phenolic acids, and stilbenes were relatively similarly distributed, while sub-group meta-analyses in polyphenol subclasses that could include five outcomes or more failed to show significant benefits for *Ginkgo biloba*, flavanol and soy isoflavones. Here, probability of positivity bias was markedly reduced, indicating a higher confidence in the results (though note the considerably smaller number of studies included in the sub-analyses). Some support for the expected significant positive effects were found for flavonoids, anthocyanins and resveratrol. In addition, within the polyphenol subclasses, efficiency was sometimes dependent on study dosage. For example, it can be speculated that the dosages in the studies administering *Ginkgo biloba* extract, namely between 38.4 and 57.6 mg flavonoids per day, were too low to affect memory. The polyphenol dosages for *Pinus radiata* bark extract and spearmint extract were higher, namely 768 mg flavonoids per day and 216 mg polyphenols per day, respectively. Also, inter-individual differences in polyphenol absorption, metabolism, and excretion might be other reasons of diverging study results. For instance, Bensalem et al. (31) found that the group with the highest EM decline excreted significantly higher amounts of phenolic metabolites while this group consumed fewer polyphenols than the groups with better performing EM. Possibly, polyphenol supplementation in the group with the highest amount of cognitive decline compensates for the lower intake and higher excretion, leading to improved memory performances in people with more severe memory decline. However, as sample characteristics, exact polyphenol formulas and dosages varied considerably between studies, as well as metabolomics of polyphenols were seldomly reported, results are difficult to harmonize in this regard. A longitudinal observational study in >2,500 older adults also pointed toward the possibility that the effects of polyphenol subclass and molecule may be per se different but also with regard to their impact on cognitive domain: Here, higher intake of e.g., catechins and flavonols were related to both higher verbal memory performance after a period of more than 10 years, while this also correlated with lower performance in tasks on executive function (99). However note that as most extracts in the reviewed studies provided a mixture of polyphenolic molecules, a detailed cause-response investigation with regard to memory function seemed difficult. Future studies should incorporate blood-based biomarkers of individual polyphenol availability and metabolism, to further understand potential differential effects of polyphenol subclasses on cognitive performance.

### Impact of Age, Pathologies, and Gender

Mild-to-moderate Alzheimer's disease patients were only studied in a small proportion of RCTs, and presenting on global cognitive test results such as the MMSE instead of WM/EM-focused subtests prevented from adding most of these to the meta-analyses. This underlines the difficulty to differentiate results between non-pathological and pathological aging populations. Considering results of subgroup meta-analyses, however, polyphenols seemed to exert positive effects in MCI and AD or vascular dementia patients on WM, but not significantly on EM, in contrast to meta-analyses in healthy groups. In addition, studies in older women only could not show significant improvements on average, yet, this might be also due to a lack of effects of isoflavonols, as this was the polyphenol used in most of these studies. Another interpretation is that both *Ginkgo biloba* and soy isoflavones had attracted probably the most interest in the last decades as a dietary supplement to combat cognitive decline, potentially due to certain hypes around alternative, nutrition-guided medicine approaches and to the estrogenic action attributed to isoflavones. Thus, these polyphenols were studied most extensively in the literature also with larger, longer-term and high-quality RCTs, leading to a more balanced reporting and resulting (partly) null findings, which would fit to some of the subgroup meta-analyses.

Still, differences in sample age, pathology and gender between studies could have contributed to mixed results. For example, positive effects of *Pinus radiata* bark and spearmint extracts were found in relatively young age, while no effects were seen in samples that were on average 10–25 years older. Moreover, on study that could not demonstrate any positive effect, meaning no objective or subjective effect, of a polyphenol-rich extract on memory, was performed in the oldest sample (81). This was a sample consisting of patients with dementia or age-associated memory impairment. Consequently, it is possible that polyphenols are more effective in relatively young and unaffected samples, but less effective in older patient samples. In addition, menopause in women is associated with an increased risk for developing metabolic syndrome, known for disturbances in glucose metabolism (100). It has been hypothesized that the negative consequences of cardiovascular risk factors on memory performance, for example higher glucose levels, can be compensated by certain polyphenols such as resveratrol administration due to regulation of glucose metabolism and insulin sensitivity (101), rendering a possible efficacy of polyphenols in postmenopausal women likely. In a previous study in older adults, we found that resveratrol lowered glycated hemoglobin A1c in blood, a long-term marker of glucose, which was associated with improved functional connectivity of the hippocampus with the medial prefrontal cortex and eventually memory retention (84). Also, reductions in insulin after cocoa flavonols correlated with increases in memory performance, supporting a potential link between polyphenols and insulin sensitivity as beneficial mechanism (59).

However, samples were often not fully characterized with regard to cardiovascular and metabolic health as well as brain diseases, i.e., (clinical) blood and neuroimaging biomarker analysis has not always been performed. Future studies with larger, more diverse samples and sensitive monitoring of cardiovascular, metabolic and beginning brain pathologies are needed to better understand the impact of age, (beginning) pathologies, and gender on polyphenol action.

### Underlying Mechanisms

Several mechanisms have been proposed that might underly the beneficial effects of polyphenols on brain aging. These include anti-oxidative and anti-inflammatory mechanisms and improvements in cardiovascular health such as lower blood pressure and better insulin sensitivity, which are all related to better brain structure and function. *Pinus radiata* bark extract, for example, has been discussed to inhibit oxidation, to reduce systolic blood pressure and to modify signaling in the brain due to the ability of polyphenol metabolites to cross the blood-brain barrier (69). The reduction of oxidation byproducts in the hippocampus has been suggested as a possible mechanism of spearmint extract (45). Moreover, a recent meta-analysis of carotenoids, known to exert anti-oxidant properties *in vitro* and *in vivo*, provides evidence for a positive effect of carotenoids on improving cognitive performance in middle-aged and older adults, further supporting the hypothesis of a causal role of antioxidant actions in the beneficial effect of plant-derived nutrients on brain health (102). A reduction in pro-inflammatory markers has for instance been observed after anthocyanin-rich supplements, thereby improving hippocampus-dependent memory performance. Kent et al. (13) did however not find altered levels of inflammatory markers in blood, although these alterations might have been undetectable due to disease progression. Yet, significantly lower systolic blood pressure was found by Kent et al. (13) and Whyte et al. (82) after polyphenol administration, which might be a consequence of reduced inflammatory markers and could relate to better memory functioning. For more details on suggested mechanistic pathways linking specific nutrients to cognitive function in aging please see other reviews [e.g., (4, 16, 102–104)].

### Limitations

Several limitations of this review should be considered. First, several studies could not be added to our meta-analysis due to lack of raw values, specific information or unplausibility in the given tables, limiting interpretability. Second, only one search base was included (PubMed), due to the limited access to Cochrane and EMBOS library, leading to a risk of omitting published study results. In addition, the results of the different types of polyphenols on WM and EM should be interpreted with care, since the number of studies per polyphenol subclass or extract was still relatively small. Thirdly, a wide variety of memory tests were used, and we cannot exclude that potential arbitrary differences in the classification to WM/EM category per author may have emerged (105). Only a few studies reported test validity and low construct validity might lead to drawing incorrect conclusions from the results. These factors might have contributed to the large statistical heterogeneity observed in the meta-analyses, and reported findings given in the systematic review may not withstand a tight control of type-1 error in the individual studies, in line with the often detected probable positivity bias. In addition, we did not evaluate included studies on study quality e.g., using established tools such as the GRADE or Cochrane risk of bias (106) due to the sheer number of studies screened, which could have revealed for example a lack of blinding or inadequate statistical reporting. However, all studies incorporated a randomized clinical trial design and a sample size of *n* ≥ 20 per group, reducing the likelihood of extreme outliers. Also, doses of the different polyphenols were barely comparable and not harmonized at all. All studies reported daily-administered amounts, but the effect of a specific dose of for example isoflavone is hard to compare with the effect of the same amount of flavanol, and compliance measures often relied on self-report or capsule count once at the end of studies.

In general, effect sizes in nutrition sciences and lifestyle interventions are expected to be rather small due to confounding factors in a free-living population. In contrast to other fields, lifestyle interventions have a long tradition of being pre-registered, e.g., on ClinicalTrials.gov and nowadays also on osf.io, which enables to restrict the number of *post-hoc* statistical testing and the possibility to report null or negative findings. Indeed, effect sizes in pre-registered studies were shown to be smaller in pre-registered studies compared to non-pre-registered studies (107). Therefore, the herein presented results of a significant effect for EM and WM are likely to be expected and indeed representative of the field.

Future studies should harmonize control conditions and use memory tests with high construct validity and focus on the quality of the methods. Methodological quality can be increased by for example concealment of allocation, using an intention-to-treat analysis, and measuring compliance as well as implementing memory tests that are insensitive to ceiling or test-retest effects, such as the computerized administration of the Mnemonic Similarity Task (108). Moreover, future studies should include larger samples and longer follow-up to increase power. Yet, the sample sizes and durations of the RCTs included in this review did not seem to affect the results. By including biological parameters, such as urine or blood samples, and functional and structural brain measures using e.g., magnetic resonance imaging, more insights on the mechanisms of polyphenols in improving memory might be gained. Performing longer longitudinal studies in the future could provide insight into whether the consumption of polyphenols decreases progression rates of patients with mild neurocognitive disorder to dementia.

## Conclusion

Based on reviewing 66 short- to longer-duration daily polyphenol intervention RCTs with small to large sample sizes, a beneficial effect of polyphenols on WM and EM in middle-aged to older adults may be considered small on average, according to qualitative review and a pooled meta-analysis of all available outcomes of 49 studies. The reported outcome measures largely varied and some studies of longer duration and larger sample sizes did not report any significant memory improvement after polyphenol administration. We also noted strong evidence for reporting bias and the statistical heterogeneity was considerably large between studies. Thus interpretation warrants caution and needs to be confirmed by further research. Future studies are encouraged to harmonize polyphenol formulas and doses as well as neuropsychological test methodology, and to increase sample sizes and follow-up periods. Overall, dietary supplementation studies investigating diet-effects on memory of high quality do exist, however, suffer from known limitations in the field and the problem to investigate rather small expected effects. Future studies should aim to address these challenges through rigorously implementing the advantages of open science, including data and code sharing, transparent reporting of neuropsychological methods and null/negative findings, and detailed pre-registration of RCTs, including a detailed statistical analysis plan, to increase reliability and to enable further meta-analyses and replication.

## Data Availability Statement

The original contributions presented in the study are included in the article as meta-data. Further inquiries related to effect sizes per study or other queries can be directed to the corresponding author.

## Author Contributions

KV, EM, and AVW: conceptualization and data analysis. KV and AVW: conducted literature search. KV: first draft. EM and AVW: visualization. EM, AVW, and AK: review and correction. All authors contributed to the article and approved the submitted manuscript.

## Funding

This work was supported by grants of the German Research Foundation No. WI 3342/3-1 and 209933838 CRC1052-03 A1 (AVW) and by the German Federal Environmental Foundation (EM).

## Conflict of Interest

The authors declare that the research was conducted in the absence of any commercial or financial relationships that could be construed as a potential conflict of interest.

## Publisher's Note

All claims expressed in this article are solely those of the authors and do not necessarily represent those of their affiliated organizations, or those of the publisher, the editors and the reviewers. Any product that may be evaluated in this article, or claim that may be made by its manufacturer, is not guaranteed or endorsed by the publisher.
